# Boosting CdS Photocatalytic Activity for Hydrogen Evolution in Formic Acid Solution by P Doping and MoS_2_ Photodeposition

**DOI:** 10.3390/nano12030561

**Published:** 2022-02-06

**Authors:** Junchen Liu, Haoran Huang, Chunyu Ge, Zhenghui Wang, Xunfu Zhou, Yueping Fang

**Affiliations:** 1 Key Laboratory for Biobased Materials and Energy of Ministry of Education, College of Materials and Energy, South China Agricultural University, 483 Wushan Road, Guangzhou 510642, China; exgukon@163.com (J.L.); hhr210527@163.com (H.H.); gcy199991@163.com (C.G.); 2 School of Chemistry and Chemical Engineering, Lingnan Normal University, Zhanjiang 524048, China; 3 Guangdong Laboratory for Lingnan Modern Agriculture, 483 Wushan Road, Guangzhou 510642, China

**Keywords:** photocatalytic H_2_ evolution, CdS, phosphorus doping, cocatalyst, in situ photodeposition, noble-metal-free cocatalyst

## Abstract

Formic acid is an appealing hydrogen storage material. In order to rapidly produce hydrogen from formic acid under relatively mild conditions, high-efficiency and stable photocatalytic systems are of great significance to prompt hydrogen (H_2_) evolution from formic acid. In this paper, an efficient and stable photocatalytic system (CdS/P/MoS_2_) for H_2_ production from formic acid is successfully constructed by elemental P doping of CdS nanorods combining with in situ photodeposition of MoS_2_. In this system, P doping reduces the band gap of CdS for enhanced light absorption, as well as promoting the separation of photogenerated charge carriers. More importantly, MoS_2_ nanoparticles decorated on P-doped CdS nanorods can play as noble-metal-free cocatalysts, which increase the light adsorption, facilitate the charge transfer and effectively accelerate the hydrogen evolution reaction. Consequently, the apparent quantum efficiency (AQE) of the designed CdS/P/MoS_2_ is up to 6.39% at 420 nm, while the H_2_ evolution rate is boosted to 68.89 mmol·g^−1^·h^−1^, which is 10 times higher than that of pristine CdS. This study could provide an alternative strategy for the development of competitive CdS-based photocatalysts as well as noble-metal-free photocatalytic systems toward efficient hydrogen production.

## 1. Introduction

With the rapid development of modern industry and inexhaustible energy consumption, hydrogen energy has attracted tremendous attention as a clean and efficient energy source. In order to use hydrogen energy in a scalable and convenient manner, hydrogen storage and transportation are of vital importance. Formic acid is an ideal material for hydrogen storage due to its high hydrogen proportion [1], low cost, low toxicity and high stability [2]. In order to extract hydrogen from formic acid for better utilization, traditional methods have adopted gold [3], silver [4], palladium [5] and other precious metals as catalysts to degrade formic acid to produce hydrogen under high temperature conditions. Such expensive catalysts and harsh reaction conditions limit the further development and application of formic acid in hydrogen storage.

Recently, the photocatalytic H_2_ evolution from formic acid by directly using clean solar energy has intrigued widespread attention [6], in terms of the advantages of not only being eco-friendly and the abundance of solar energy, but also the fact that relatively mild conditions and pollution-free products are involved in the photocatalytic process. Photocatalysis was first discovered in the 1970s by Fujishima and Honda [7]. Since then, a large number of photocatalysts have been continuously developed, and some of them have been used for photocatalytic H_2_ evolution from formic acid, such as g-C_3_N_4_ [8], SrTiO_3_ [9], ZnO [10], ZnS [11], CdS [12], etc. Among numerous semiconductor photocatalysts, CdS can be excited by visible light due to its band gap of about 2.4 eV, which makes it a promising photocatalytic candidate [13]. However, it has been proved that CdS always suffer from fast recombination of photogenerated charge carriers and severe photocorrosion, which seriously block the improvement of the photocatalytic efficiency of CdS photocatalysts [14,15]. In order to improve the light utilization and photocatalytic efficiency of CdS, commonly used solutions include element doping [16,17], cocatalyst loading [18,19,20,21] and the construction of heterojunctions [19,22,23,24]. Especially, elemental phosphorus (P) has been introduced into the CdS lattice to reduce the free energy of H_2_ adsorption and enhance its visible light absorption and corrosion resistance [24,25,26]. Moreover, Huang et al. has demonstrated that gradient doping of P in CdS can generate an oriented built-in electric field, thus improving the efficiency of charge extraction [25]. In addition to element doping, the loading of appropriate cocatalysts is also a very effective way to improve the photocatalytic efficiency of CdS. Generally speaking, after loading a suitable cocatalyst, the photogenerated electrons or holes of photocatalyst will quickly migrate to the cocatalyst for photocatalytic reactions, thereby greatly promoting the separation rate of photogenerated charge carriers [27]. Furthermore, the introduction of desirable cocatalysts can reduce the energy barrier required for redox reactions on the photocatalyst surfaces [27]. Noble metals, such as platinum [28] and gold [29], have been widely used in the construction of efficient photocatalytic systems because of their good cocatalytic activity, high conductivity and acceptable stability. Due to the scarcity of noble metals and the high cost, cocatalysts without noble metals are preferred so as to build a low-cost photocatalytic system that can be widely promoted. For example, Co_x_O_y_ [30,31], MoS_2_ [19], MoN [32], Ni_x_P [33], Co_x_P [6], MoP [34], Co_3_C [35], Ni_3_C [36], CoFe_2_O_4_ [37], and NiFe_2_O_4_ [38,39] have been widely studied and made encouraging progress. Among them, 2D structured MoS_2_ has excellent H_2_ evolution reaction activity and high specific surface, which make it as very prospective cocatalyst. On the basis of a few studies on CdS/MoS_2_, it has been proved that the charge separation rate of CdS can be enhanced by loading with MoS_2_ and the corresponding photocatalytic performance of H_2_ evolution from formic acid can be also improved [40]. However, the photocatalytic H_2_ evolution rate of formic acid to H_2_ in the reported CdS/MoS_2_ systems are still not ideal. In order to release hydrogen energy from formic acid more quickly, it is necessary to establish a photocatalytic system based on CdS and MoS_2_ with higher photocatalytic activity that has the ability of rapid photocatalytic H_2_ production from formic acid. Yang et al. has demonstrated that P-doped CdS with MoSe_2_ cocatalysts can greatly improve the performance of photocatalyst [41]. Evidently, elemental P doping combined with MoS_2_ loading could be potentially effective to improve the photocatalytic activity of CdS for H_2_ evolution.

Based on above considerations, in this work, CdS nanorods are doped with elemental P (denoted as CdS/P), and then loaded with MoS_2_ on the surfaces by in situ photodeposition (denoted as CdS/P/MoS_2_), aiming to prepare an efficient and stable photocatalytic system of H_2_ evolution from formic acid. The as-prepared CdS/P/MoS_2_ system shows significantly enhanced photocatalytic activity and stability compared with pristine CdS. The apparent quantum efficiency (AQE) of the CdS/P/MoS_2_ is as high as 6.39% at 420 nm, while the H_2_ evolution rate is up to 68.89 mmol·g^−1^·h^−1^, which is 10 times higher than that of pristine CdS.

## 2. Materials and Methods

### 2.1. Preparation of the CdS Nanorods

A total of 4.627 g of cadmium nitrate tetrahydrate, 3.425 g of thiourea, and 65 mL of ethylene diamine were added to a 100 mL hydrothermal reaction kettle. After electromagnetic stirring for 30 min, the reaction system was sealed, heated to 160 °C, and kept for 48 h. After the hydrothermal reactor was naturally cooled to room temperature, the reaction product was centrifuged, washed with 95% ethanol for three times, and dried at 60 °C for 24 h. The received product was CdS nanorods.

### 2.2. Phosphating of CdS Nanorods

A total of 500 mg of sodium hypophosphite (NaH_2_PO_2_) and 500 mg of CdS nanorods were grinded together evenly. Then, the mixture was calcined at 300 °C for 2 h in a slow N_2_ flow. Finally, the calcined mixture was washed with deionized water and dried, and CdS/P was obtained.

### 2.3. Preparation of CdS/P/MoS_2_ and CdS/MoS_2_

CdS/P/MoS_2_ and CdS/MoS_2_ were prepared by in situ photodeposition. A total of 50 mg of CdS/P or CdS was mixed with 100 mL deionized water, followed by ultrasonic dispersion for 20 min. The uniformly dispersed suspension was then added with a certain amount of ammonium tetrathiomolybdate solution. N_2_ was continuously injected into the suspension and stirred for 30 min to remove oxygen from the suspension. After injection, the suspension was continuously irradiated with visible light for 1 h (300 W xenon lamp installed with 400 nm cut-off filter, input voltage of 220 V, current of 20 A, Beijing Perfectlight, PLS-SXE300D). After the illumination, the precipitation was recovered by filtration. After being washed with deionized water for three times, and being washed with 95% ethanol, the precipitates were dried in an oven at 60 °C for 12 h to obtain CdS/P/MoS_2_ or CdS/MoS_2_, respectively.

### 2.4. Performance Test of Photocatalytic Hydrogen Evolution

A total of 10 mg of the photocatalyst prepared above were taken and dispersed in 80 mL deionized water by ultrasonic. After the ultrasonic dispersion, 20 mL formic acid was added into the suspension, and N_2_ was continuously injected for 30 min to remove the dissolved oxygen in the suspension. After the deoxygenated suspension was irradiated by xenon lamp (installed with 400 nm cut-off filter, input voltage of 220 V, current of 20 A, Beijing Perfectlight, PLS-SXe300D), the generated H_2_ was detected by Techcomp GC 7900 installed with a TCD detector. The apparent quantum efficiency (AQE) was calculated by following formula [2,42,43]:

$$\mathrm{AQE}=\frac{\mathrm{Number}\;\mathrm{of}\;\mathrm{evolved}\;H_{2}\;\mathrm{molecules}\;\times \;2}{\mathrm{Number}\;\mathrm{of}\;\mathrm{incident}\;\mathrm{photons}}\times 100\%$$

### 2.5. Characterizations

Powder X-ray diffraction (XRD) measurements were recorded on a Rigaku D/MAX-2500 diffractometer with monochromatized Cu Kα radiation (λ = 0.15418 nm). TEM, HRTEM and EDX mapping were recorded on the FEI Glacios Cryo-TEM equipped with an energy dispersive X-ray (EDX) spectroscope, operating at 200 kV. X-ray photoelectron spectra (XPS) were recorded on a VG ESCALAB 250Xi spectrometer, using a standard Al Kα X-ray source (300 W) and an analyser pass energy of 20 eV. Samples were mounted using double-sided adhesive tape and the binding energies were referenced to the C (1 s) binding energy of adventitious carbon contamination taken to be 284.7 eV. UV-vis absorption spectroscopy was conducted on a Shimadzu-2501 PC spectrophotometer using an integrating-sphere accessory. Room temperature photoluminescence (PL) spectra were recorded on a PerkinElmer LS 50B fluorescence spectrophotometer with an excitation wavelength of 300 nm. The PL decay plots were conducted on an Edinburgh Instruments F980 fluorescence lifetime spectrophotometer.

### 2.6. Photoelectrochemical Measurements

The preparation process of the photoelectrode was as follows: 10 mg photocatalyst, 2 mL anhydrous ethanol, 100 μL 5 wt.% Nafion solution were mixed and performed under ultrasonic for 30 min to obtain the evenly mixed suspension. A total of 400 μL suspension was dropped onto the FTO glass substrate (2 cm × 6 cm) and dried under an infrared lamp. Then, the FTO glass base was put into the porcelain ark and placed in a tubular furnace, which was heated to 150 °C at 3 °C min^−1^ and kept for 1 h under nitrogen protection, and finally cooled naturally to room temperature. The optical electrode was prepared by cutting the FTO glass substrate into 1 cm × 2 cm with a laboratory glass cutter. CHI 650E electrochemical workstation was used to form a three-electrode test system for photoelectrochemistry tests with the above prepared photoelectrode as the working electrode, of platinum sheet as the counter electrode and Ag/AgCl as the reference electrode. A 300 W xenon lamp was adopted as the light source. The polarization curves of the electrochemical H_2_ evolution reaction, transient photocurrent response test, Mott–Schottky test and electrochemical impedance test were carried out in 0.5 M sodium sulphate solution, and the polarization curves of the electrochemical oxygen evolution reaction were tested in 1 M KOH solution. Transient photocurrent response was tested using an applied voltage of 0.4 V. The scanning speed of the polarization curves was 0.005 V·s^−1^. Electrochemical impedance tests were performed with an amplitude of 0.005 V and frequencies of 0.01–10^5^ Hz. The Mott–Schottky test was performed by an amplitude of 0.005 V and a frequency of 1000 Hz. The flat band potential was estimated using a Mott–Schottky curve via the following formula [44]:

$$\frac{1}{C^{2}}=[\frac{2}{e_{0}ɛ\cdot {ɛ}_{0}N_{d}A^{2}}]\cdot [E─E_{fb}─\frac{KT}{e}]\;$$

The potentials to the Ag/AgCl electrode were calibrated to that of the reversible hydrogen electrode (RHE) and calculated by the following formula:

$$E_{RHE}=E_{AgCl}+0.059\times pH+E_{AgCl}^{\Theta }(E_{AgCl}^{\Theta }=0.197\;V)\;$$

## 3. Results and Discussion

### 3.1. Characterization of the Composition, Structure and Morphology of the Photocatalysts

**Figure 1:**
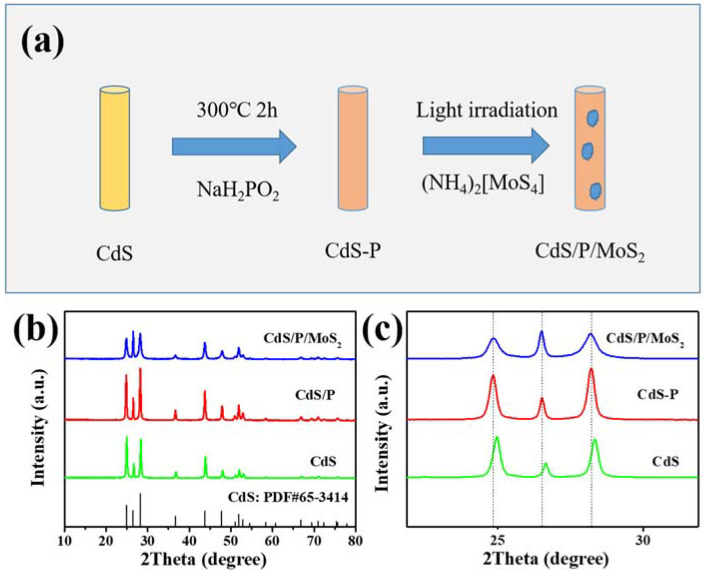
(**a**) Schematic diagram of preparation for CdS/P and CdS/P/MoS_2_; (**b**) XRD patterns of CdS, CdS/P and CdS/P/MoS_2_; and (**c**) locally enlarged XRD patterns.

As shown in Figure 1a, NaH_2_PO_2_ was used as the P source to generate PH_3_ gas at 300 °C. PH_3_ gas can react with CdS nanorods to generate CdS/P. With ammonium tetrathiomolybdate as the precursor, the reaction of loading MoS_2_ on the surface of CdS/P by in situ photodeposition is shown below:

$$\mathrm{CdS}/P\overset{h\nu }{\to }e^{-}+h^{+}\;{\left[MoS_{4}\right]}^{2-}+2e^{-}\to MoS_{2}+2S^{2-}$$

First, CdS/P produced photo-generated electrons and holes under the visible light irradiation. Then, [MoS_4_]^2−^ adsorbed on the surface of CdS/P nanorods was reduced to MoS_2_ by the photogenerated electrons transferred to the surface of the CdS/P nanorods, thereby forming CdS/P/MoS_2_. Finally, the photo-generated holes were consumed through the oxidation of water molecules.

The XRD patterns of CdS, CdS/P, CdS/P/MoS_2_ are shown in Figure 1b. All diffraction peaks of CdS/P and CdS/P/MoS_2_ can be well attributed to the characteristic peaks of CdS (PDF#:65-3414). As shown in Figure 1c, compared with the diffraction peaks of CdS, three peaks of CdS/P and CdS/P/MoS_2_ shift to lower angles, suggesting that elemental P was successfully doped into the lattices of CdS [26,45,46]. As shown in Figure 1c, the intensity ratio of the three diffraction peaks of CdS is 2.7 (100):1.0 (002):2.6 (101), while the intensity ratio of the three diffraction peaks of CdS/P is 2.0:1.0:2.3. Compared to CdS, the peak intensity ratio of the (100) plane of CdS/P is decreased, which may affect the photocatalytic performance. Qiao et al. has demonstrated that the (100) plane of CdS benefits its photocatalytic activity [47]. Due to the low content of MoS_2_, no diffraction peaks belonging to MoS_2_ are observed in CdS/P/MoS_2_. Compared with those of CdS/P, the diffraction peak intensities of the (100) crystal plane at 24.84° and the (101) crystal plane at 28.22° for CdS/P/MoS_2_ have a slight decrease, indicating that the in situ photodeposition of MoS_2_ is selective.

**Figure 2:**
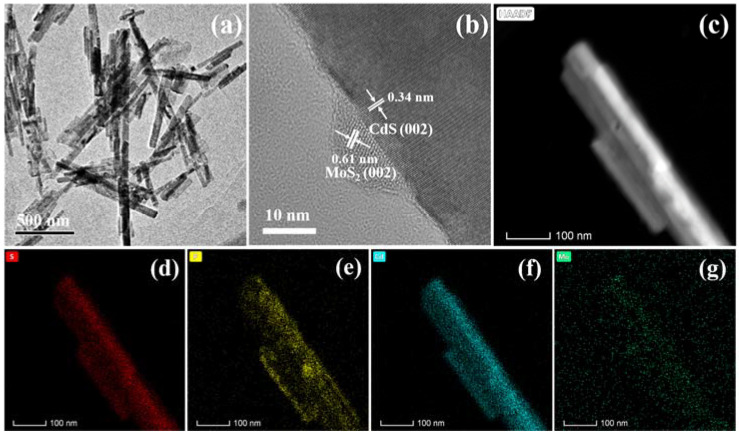
Characterization of CdS/P/MoS_2_: (**a**) TEM image; (**b**) HRTEM image; (**c**) HAADF STEM image and corresponding elemental mappings of (**d**) S; (**e**) P; (**f**) Cd; (**g**) Mo.

TEM observation showed that CdS/P/MoS_2_ still maintained the basic nanorod morphology as CdS and CdS/P (Figure 2a). MoS_2_ loading on the surface of CdS/P/MoS_2_ can be viewed by HRTEM (Figure 2b). It can be seen from Figure 2b that there are typical lattice fringes of the (002) plane with a width of 0.34 nm of CdS [48] in the middle of the nanorod, and the lattice fringes of the (002) plane with a width of 0.61 nm of MoS_2_ [48,49] were attached to the outer edges of the nanorods. Element mappings (Figure 2c) also prove that Cd, S, P and a small amount of Mo are evenly distributed throughout the nanorods.

**Figure 3:**
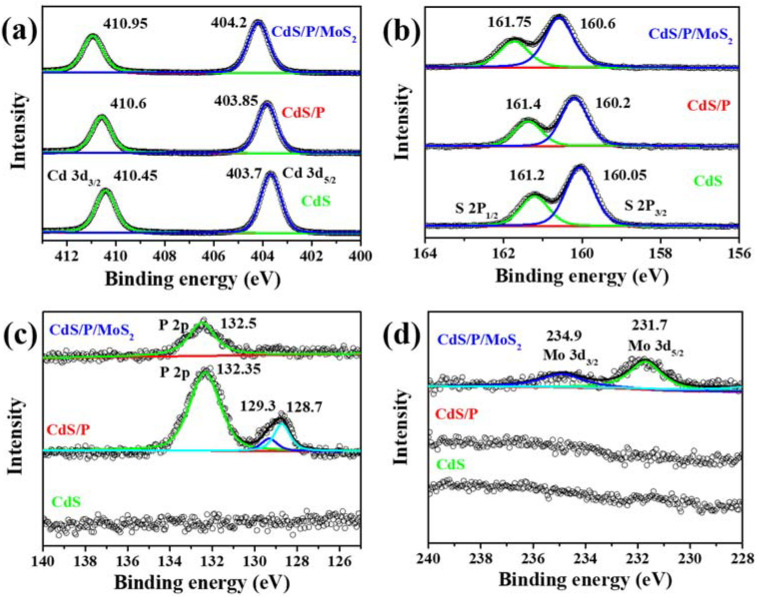
High resolution XPS spectra of CdS, CdS/P and CdS/P/MoS_2_: (**a**) Cd 3d; (**b**) S 2p; (**c**) P 2p and (**d**) Mo 3d.

The surface composition of CdS/P/MoS_2_ was further analyzed by XPS and shown in Figure 3. Observing from Figure 3a, the peaks at 410.45 and 403.7 eV in CdS correspond to Cd 3d_3/2_ and Cd 3d_5/2_, which prove that Cd exists in the state of Cd^2+^ [50]. It can be concluded from Figure 3b that the peaks at 161.2 and 160.05 eV in CdS correspond to S 2P_1/2_ and S 2P_3/2_, which prove that S exists in the state of S^2−^ [50]. When CdS was doped with phosphorus, the binding energy of Cd and S are almost unchanged, so it can be seen that phosphorus doping has no obvious effect on the chemical structure of CdS. As can be observed from Figure 3c, the peaks at 132.35 eV in CdS/P correspond to P 2p, which proves that P is successfully doped into CdS. The peaks at 129.3 and 128.7 eV demonstrate that some of P atoms exist in the form of zero-valent phosphorus [26]. In view of the integral area of the peaks, and atomic sensitivity factors, the atomic percentages of Cd, S, doped P and zero valence P of CdS/P are calculated to be 44.3 at.%, 44.0 at.%, 9.6 at.% and 2.1 at.%, respectively. After MoS_2_ loading, the peaks of Cd, S and P in CdS/P/MoS_2_ move to the direction of high binding energy, which indicates that a heterojunction is formed between CdS/P and MoS_2_ and is favorable for the charge transfer of photogenerated electrons from CdS/P to MoS_2_ [49]. It is worth noting that there is no zero-valent phosphorus in CdS/P/MoS_2_ after in situ photodeposition. This is because zero-valent phosphorus is unstable and is more likely to be oxidized during in situ photodeposition. However, P 2p still exists at 132.5 eV, which proves the stable existence of P doping. As shown in Figure 3d, there are two tiny peaks at 234.9 and 231.7 eV, corresponding to Mo 3d_3/2_ and Mo 3d_5/2_, respectively, indicating that Mo element exists in the form of Mo^4+^ [49]. Based on the integral area of the peaks, and atomic sensitivity factors, the atomic percentages of Mo, Cd, S, and P in CdS/P/MoS_2_ were calculated to be 0.6 at.%, 47.1 at.%, 48.4 at.%, and 3.9 at.%, respectively. The results of XPS further confirmed the coexistence of doped P and MoS_2_ in CdS/P/MoS_2_.

### 3.2. The Performances of the Photocatalysts for Photocatalytic H_2_ Evolution

**Figure 4:**
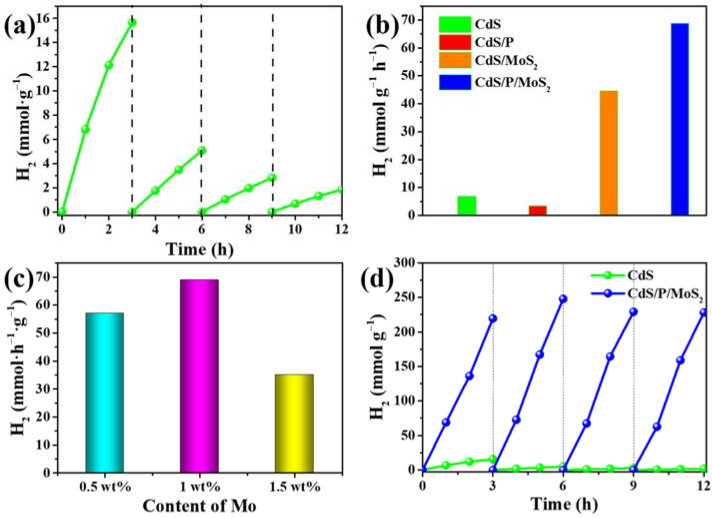
Photocatalytic H_2_ evolution in formic acid solution under visible light irradiation: (**a**) the stability test of CdS for H_2_ evolution; (**b**) the average rate of H_2_ evolution over CdS, CdS/P, CdS/MoS_2_ and CdS/P/MoS_2_; (**c**) the average rate of H_2_ evolution over CdS/P loaded with different content of Mo; (**d**) the CdS and CdS/P/MoS_2_ stability tests of H_2_ evolution.

The activities of the photocatalysts for photocatalytic H_2_ evolution from formic acid solution were evaluated under visible light. As shown in Figure 4a, the photocatalytic activity and stability of pure CdS nanorods are very poor. Its activity of H_2_ evolution from formic acid solution decayed rapidly within 12 h.

**Figure 5:**
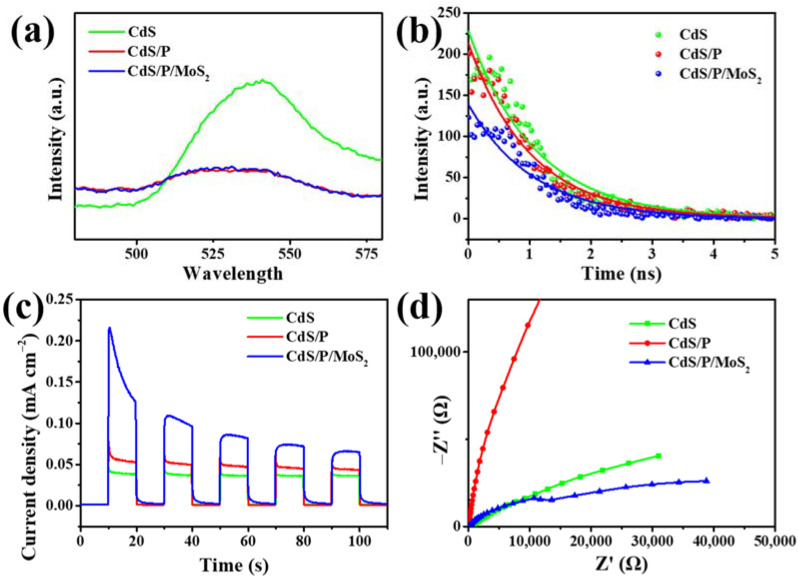
Performance tests of CdS, CdS/P and CdS/P/MoS_2_: (**a**) photoluminescence (PL) spectra; (**b**) transient PL spectra; (**c**) transient photocurrent response curves; (**d**) electrochemical impedance spectra (EIS).

**Figure 6:**
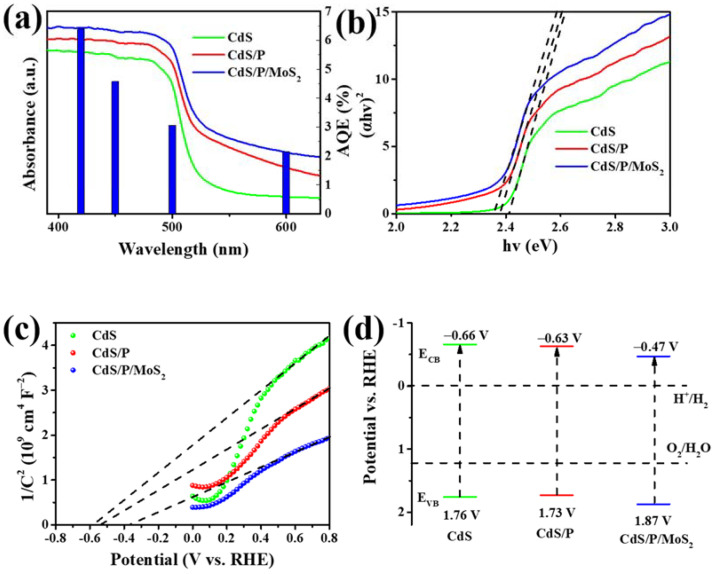
Characterization of CdS, CdS/P and CdS/P/MoS_2_: (**a**) UV-vis diffused reflectance spectra (DRS) and AQE of CdS/P/MoS_2_; (**b**) Corresponding Tauc plots of DRS; (**c**) Mott–Schottky (MS) plots and (**d**) Schematic band structure diagrams.

Figure 4b shows the H_2_ evolution rate of CdS, CdS/P, CdS/MoS_2_ and CdS/P/MoS_2_ under visible light irradiation. The H_2_ evolution rate of pure CdS nanorods is 6.8 mmol g^−1^ h^−1^. However, the rate of H_2_ evolution for CdS/P decreased (3.4 mmol g^−1^ h^−1^) while P was doped. In CdS/P, a small amount of zero-valent P covers the surface of the catalyst, resulting in the attenuation of the (100) plane (Figure 1c) and the increase in the interfacial resistance (Figure 5d). This could explain the decrease in the catalytic performance of CdS/P. The in situ photodeposition of MoS_2_ (noting that the theoretical content of Mo is 1%) on the surface of CdS significantly increased the rate of H_2_ evolution to 44.6 mmol g^−1^ h^−1^, indicating that MoS_2_ is indeed an excellent H_2_ evolution cocatalyst. When MoS_2_ (the theoretical content of Mo is still 1%) was loaded onto the surface of CdS/P, the rate of H_2_ evolution was further increased to 68.9 mmol g^−1^ h^−1^, indicating that P doping and MoS_2_ loading could synergistically promote the photocatalytic activity of CdS for H_2_ production. In order to find out the optimal doping amount of P, the effect of different NaH_2_PO_2_ dosage on the hydrogen production performance of CdS/P/MoS_2_ was evaluated (Figure S1). When the dosage of NaH_2_PO_2_ is 500 mg, that is, the mass ratio of NaH_2_PO_2_ to CdS is 1:1, the catalyst has the best performance. As shown in Figure 4c, CdS/P loaded with MoS_2_ (1 wt.% of Mo) shows the highest H_2_ evolution rate of 68.9 mmol g^−1^ h^−1^ in the formic acid solution, indicating that the theoretical Mo loading (1 wt %) is the best. The photocatalytic hydrogen production performance of CdS loaded with different MoS_2_ were also tested, and the optimal loading is consistent with CdS/P/MoS_2_ (Figure S2a). In the stability test of CdS/MoS_2_, the performance of CdS/MoS_2_ was slightly weakened after 12 h photocatalytic hydrogen production test, though its stability was still greater than that of CdS (Figure S2b). The results show that the elaborate loading of MoS_2_ is beneficial to improve the photocatalytic stability of CdS. Figure 4d shows the photocatalytic stabilities of CdS and CdS/P/MoS_2_ in the formic acid solution. After four cycles of testing within 12 h, the rate of H_2_ evolution for CdS/P/MoS_2_ did not decrease significantly. With regard to pure CdS nanorods, it can be seen from Figure 4a that only 12.5% of the initial photocatalytic activity was retained after four cycles within 12 h. Evidently, the stability of CdS/P/MoS_2_ after P doping and in situ photodeposition of MoS_2_ was greatly improved compared with that of CdS. The excellent stability of CdS/P/MoS_2_ can also be confirmed by XRD and TEM characterization (Figure S3, see Supplementary Materials for details) and XPS spectra (Figure S4). Figure S3 demonstrated that the exact structure of CdS/P/MoS_2_ does not change significantly before and after the photocatalytic reaction for 12 h. In addition, as shown in Figure 6a, the apparent quantum efficiencies of CdS/P/MoS_2_ at 420 nm, 450 nm, 500 nm and 600 nm are 6.39%, 4.57%, 3.05%, and 2.14%, respectively. The photocatalytic performances of CdS/P/MoS_2_ to degrade formic acid for hydrogen production were compared with those of the recently reported photocatalysts in Table S1 [2,6,12,51,52,53,54,55,56,57], which indicates the application potential of P-doping and MoS_2_ engineered CdS nanorods for the photocatalytic decomposition of formic acid. 

### 3.3. The Mechanism of Photocatalytic H_2_ Evolution in Formic Acid Solution

In general, broadening the light absorption range of photocatalysts can increase the amount of photogenerated charges, which are beneficial for subsequent photocatalytic redox reactions. It can be seen from Figure 6a that the light absorption band of CdS extends from the ultraviolet region to ~530 nm. After P doping, the absorption of CdS/P in the visible light range increases significantly, indicating that the introduction of the P element improves the light absorption capacity of CdS [26,58]. Moreover, the light absorption of CdS/P/MoS_2_ after MoS_2_ photodeposition is significantly enhanced from wavelength 300 nm to 800 nm, which is attributed to the effective light absorption by MoS_2_ [40].

The band gaps of CdS, CdS/P, and CdS/P/MoS_2_ were calculated by the Kubelka–Munk method based on the absorption spectrum [40]. As shown in Figure 6b, the value of the band gap was obtained by extrapolating the tangents at (αhν)^2^ = 0 when plotting (αhν)^2^ versus hν, where hν is the photon energy and α is the absorption coefficient. P doping narrows the band gap of CdS to 2.36 eV compared to CdS (2.42 eV). Additionally, the band gap of CdS/P/MoS_2_ is 2.34 eV. Evidently, CdS/P/MoS_2_ has a better light-harvesting capability to boost the photogenerated charge generation for the H_2_ evolution reaction in the formic acid solution.

The Mott–Schottky test results can confirm the band locations and help to resolve the charge migration paths in CdS/P/MoS_2_. As can be seen from the results (Figure 6c), the slope of CdS, CdS/P and CdS/P/MoS_2_ curves is positive, which proves that the three samples own n-type semiconductor characteristics [59]. As to the MS curve, the value of the X-axis intercept of the tangent line in the linear part is the flat band potential (V_fb_) of the sample. According to Figure 6c, the flat-band potential of CdS is −0.56 V vs. RHE, while the flat-band potential of CdS/P is −0.53V vs. RHE, and the flat-band potential of CdS/P/MoS_2_ is −0.37V vs. RHE. By comparing the flat band potential of the three samples, it can be found that the flat band potential of CdS/P is more positive than that of CdS, which means that the doping of P leads to the downward shift of the conduction band bottom in CdS/P [26,59]. Compared with CdS/P, the conduction band bottom of CdS/P/MoS_2_ moves down further, which is due to the more positive feature of the Fermi level of MoS_2_ compared with CdS/P. When a close contact is formed between CdS/P and MoS_2_, the electrons transfer from CdS/P to MoS_2_, resulting in band bending, which makes the Fermi level reach a new equilibrium [26,60]. Based on the above flat band potential data and band gap width, in combination with the rule that the conduction band of N-type semiconductor is more negative than the flat band by about −0.1 V, we can conclude the following band structure diagram. As can be seen from Figure 6d, the photogenerated electrons tend to transfer to MoS_2_, and the photogenerated holes tend to transfer to the surface of CdS/P. The above results indicate that the constructed CdS/P/MoS_2_ photocatalytic system can promote the charge separation and improve the photocatalytic efficiency.

In order to further characterize the charge transfer and separation ability of CdS/P/MoS_2_, optical and photoelectrochemical tests were performed. Photoluminescence (PL) spectroscopy can be used to test the separation and recombination tendency of photogenerated charge carriers. As can be seen in Figure 5a, CdS exhibits a strong fluorescence emission peak at around 540 nm, while the fluorescence intensity of CdS/P and CdS/P/MoS_2_ in the same region is much weaker than that of CdS. This means that the photogenerated charge carriers in CdS tend to recombine due to the structural limitation of individual CdS, while the photogenerated charge carriers in CdS/P and CdS/P/MoS_2_ tend to rapidly separate and reduce the recombination due to the change of the band structure caused by P doping and MoS_2_ loading. It should be noted that there is no obvious difference in fluorescence intensity between CdS/P and CdS/P/MoS_2_. This can prove that MoS_2_ loading does not increase the fluorescence intensity of the sample, that is, does not obviously impact the recombination of the photogenerated charges. Since P doping has greatly reduced the fluorescence emission peak that nearly disappeared, it is difficult to detect the decrease in fluorescence intensity after MoS_2_ loading. These results indicate that CdS/P and CdS/P/MoS_2_ have a higher light utilization ability than CdS, thus improving the efficiency of photocatalysis. Fluorescence lifetime tests were used for a more detailed analysis of the photogenerated charge carriers of the samples (Figure 5b). Through double exponential function fitting and calculation, the average fluorescence lifetime of the sample was calculated, as shown in Table S2. The average lifetime of CdS/P/MoS_2_ is 1.05 ns, which is much lower than that of CdS/P (2.51 ns) and CdS (2.66 ns). This proves that CdS/P/MoS_2_ has a higher nonradiative transition rate, which is attributed to the accelerated transfer of electrons from CdS/P to MoS_2_ [58]. Ac impedance spectroscopy (Figure 5d) was further used to analyze the interfacial resistance of the photocatalysts. In the Nyquist plots, the smaller arc represents a smaller interfacial resistance, meaning that the charges can be transferred more easily from the surface of the catalysts to the solution [61]. It can be seen that the arc of CdS/P is larger than that of CdS, which could be caused by the small amount of red phosphorus attached to the surface of CdS/P, and increases the interfacial resistance between CdS/P and the solution. It is worth noting that with the loaded MoS_2_, the interface resistance of CdS/P/MoS_2_ decreases significantly compared to CdS/P, indicating that the presence of MoS_2_ is very conducive to the charge transfer in the semiconductor photocatalyst. Transient photocurrent response was used to further clarify the ability of the photocatalyst to inhibit the recombination of photogenerated electrons and holes. As shown in Figure 5c, the current density of CdS/P is significantly higher than that of CdS. Although CdS/P has a higher interfacial resistance than CdS, it is difficult for the charges on the surface of CdS/P to transfer to the solution. However, as the doping of P can change the energy band structure of the photocatalyst and improves the light response range, the photogenerated charges are more easily to be transferred from the inside of CdS/P to the surface, resulting in an increase in the photocurrent. The photocurrent of CdS/P/MoS_2_ is significantly higher than that of CdS/P. This indicates that the interfacial interaction between CdS/P and MoS_2_ leads to the rapid migration of photogenerated electrons from CdS/P to MoS_2_, and therefore CdS/P/MoS_2_ achieved better photocatalytic performance due to its smaller interfacial resistance. All above optical and photoelectrochemical tests have proved that CdS/P/MoS_2_ owns a stronger light response and enhanced charge separation efficiency.

**Figure 7:**
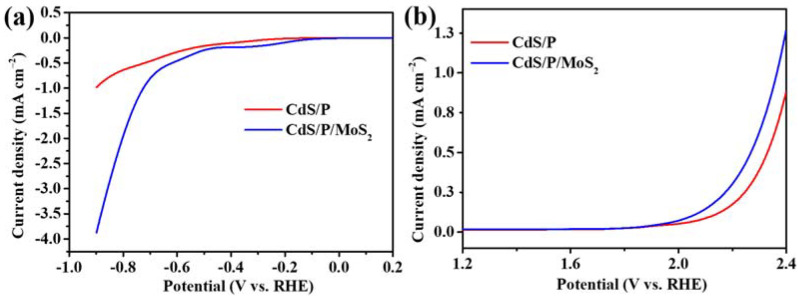
The performance tests of CdS/P and CdS/P/MoS_2_: (**a**) electrocatalytic HER polarization curves and (**b**) electrocatalytic OER polarization curves.

The effect of MoS_2_ as a cocatalyst in the photocatalytic process can be further characterized by the electrocatalytic hydrogen evolution reaction and oxygen evolution reaction, which in turn provide more clues to characterize the reactive activity of photocatalytic oxidation and reduction [62]. Figure 7 shows the electrocatalytic H_2_ evolution reaction (HER) curve and oxygen evolution reaction (OER) curves of CdS/P and CdS/P/MoS_2_ tested by linear sweep voltammetry. It can be seen that the in situ photodeposition of MoS_2_ can significantly reduce the HER and OER overpotentials at the same time. Therefore, it can be inferred that the conduction band of CdS is located in a more reductive zone compared to the composites (Figure 6d), but its H_2_ reduction rate seems to be less. This is because the kinetics of photocatalytic H_2_ evolution reaction on the surface of CdS is sluggish. The in situ photodeposited MoS_2_ cocatalyst can significantly reduce the dynamic barrier of H_2_ evolution reaction on the surfaces of CdS/P/MoS_2_ and CdS/MoS_2_. Therefore, after the loading of MoS_2_, the rate of photocatalytic H_2_ evolution for CdS/P/MoS_2_ and CdS/MoS_2_ are increased significantly.

**Scheme 1:**
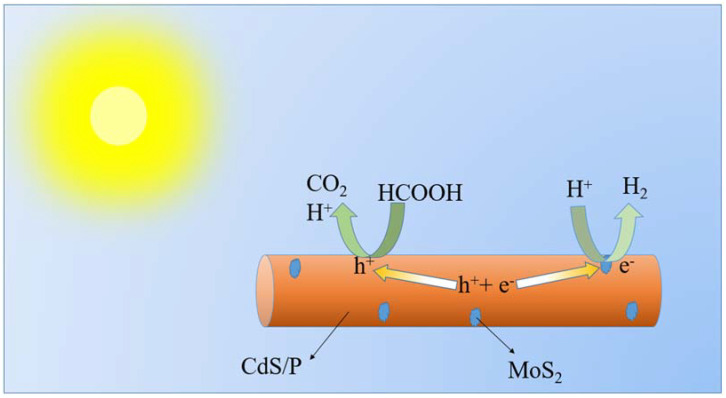
Schematic illustrations of the charge transfer behavior of CdS/P/MoS_2_, and its photocatalytic HER and OER mechanism.

Based on the above results, a reasonable mechanism of photocatalytic H_2_ evolution by CdS/P/MoS_2_ in the formic acid solution was summarized. As shown in Scheme 1, photoinduced charges are firstly generated when the photocatalyst receives light radiation. The Schottky energy barrier generated by the interfacial interaction between CdS/P and MoS_2_ can promote the transfer of the photogenerated electrons from CdS/P to the surface-loaded MoS_2_ cocatalyst. Photogenic holes remain in CdS/P and migrate to the surface. The combination of MoS_2_ as a cocatalyst with CdS/P could synergistically reduce the energy barriers of photocatalytic HER and OER, resulting in the reduction in H^+^ in the formic acid solution by photogenerated electrons in MoS_2_ to generate H_2_, while the photogenerated holes are consumed by formic acid. In summary, CdS/P/MoS_2_ photocatalyst can realize a significant efficiency in the photocatalytic H_2_ evolution in the formic acid solution.

## 4. Conclusions

In conclusion, we have demonstrated that elemental P doping combining with the in situ photodeposition of MoS_2_ can improve the photocatalytic activity and stability of CdS for H_2_ evolution from formic acid. The significantly enhanced photocatalytic activity of the CdS/P/MoS_2_ system resulted from the improved visible light absorption, the fast separation of photogenerated charge carriers and more exposed active sites of the surface engineered CdS for hydrogen evolution. The present study could provide a pathway to construct cost-efficient and durable noble-metal-free photocatalytic systems for H_2_ evolution from formic acid.

## Data Availability

Data can be available upon request from the authors.

## Supplementary Materials

The following supporting information can be downloaded at: www.mdpi.com/article/10.3390/nano12030561/s1. Figure S1: The average rate of H_2_ evolution over CdS/P/MoS_2_ synthesized from different dosage of NaH_2_PO_2_; Figure S2: The average rate of H_2_ evolution over CdS/MoS_2_ loaded with different contents of Mo (a) and stability tests of CdS and CdS/MoS_2_ (b); Figure S3: The characterization of CdS/P/MoS_2_ after recycling tests: (a) HRTEM; (b) XRD; Figure S4: The high resolution XPS spectra of CdS/P/MoS2 after a photocatalytic run: (a) Cd 3d; (b) S 2p; (c) P 2p and (d) Mo 3d; Table S1: Comparison of the photocatalytic H_2_ production activities of different photocatalyst; Table S2: Exponential decay-fitted parameters of fluorescence lifetime for CdS, CdS/P and CdS/P/MoS_2_.
