# Supplementary material for: Boosting CdS Photocatalytic Activity for Hydrogen Evolution in Formic Acid Solution by P Doping and MoS2 Photodeposition
Source: Nanomaterials (Basel). 2022 Feb 6;12(3):561. doi: 10.3390/nano12030561 (PMC8839047; doi:10.3390/nano12030561)
Supplement: Supplementary file 1 [file nanomaterials-12-00561-s001.zip › nanomaterials-1547938-supplementary.pdf]

Supplementary Materials

Boosting CdS Photocatalytic Activity for Hydrogen Evolution in Formic Acid Solution by P Doping and MoS_2 Photodeposition

Junchen Liu [1], Haoran Huang [1], Chunyu Ge [1], Zhenghui Wang [1,*], Xunfu Zhou [2,*] and Yueping Fang [1,3,*]

[1] Key Laboratory for Biobased Materials and Energy of Ministry of Education, College of Materials and Energy, South China Agricultural University, 483 Wushan Road, Guangzhou 510642, China; exgukon@163.com (J.L.); hhr210527@163.com (H.H.); gcy199991@163.com (C.G.)
[2] School of Chemistry and Chemical Engineering, Lingnan Normal University, Zhanjiang 524048, China
[3] Guangdong Laboratory for Lingnan Modern Agriculture, 483 Wushan Road, Guangzhou 510642, China
* Correspondence: zhwang@scau.edu.cn (Z.W.); zxf776932260@163.com (X.Z.); ypfang@scau.edu.cn (Y.F.)

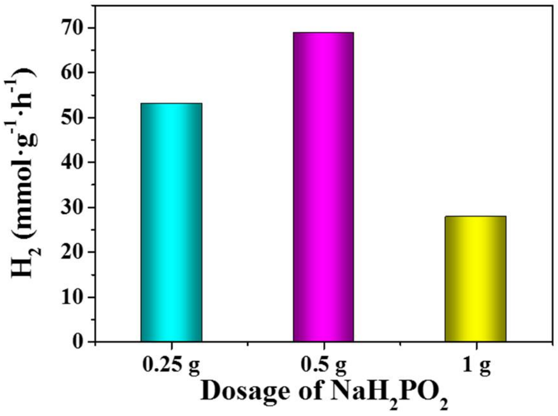

Figure S1. The average rate of H_2 evolution over CdS/P/MoS_2 synthesized from different dosage of NaH_2PO_2,.

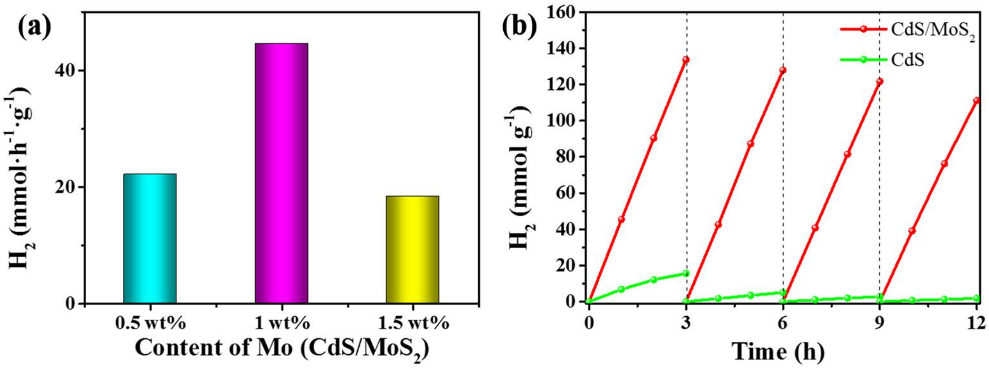

Figure S2. The average rate of H_2 evolution over CdS/MoS_2 loaded with different content of Mo.(a) and stability tests of CdS and CdS/MoS_2 (b).

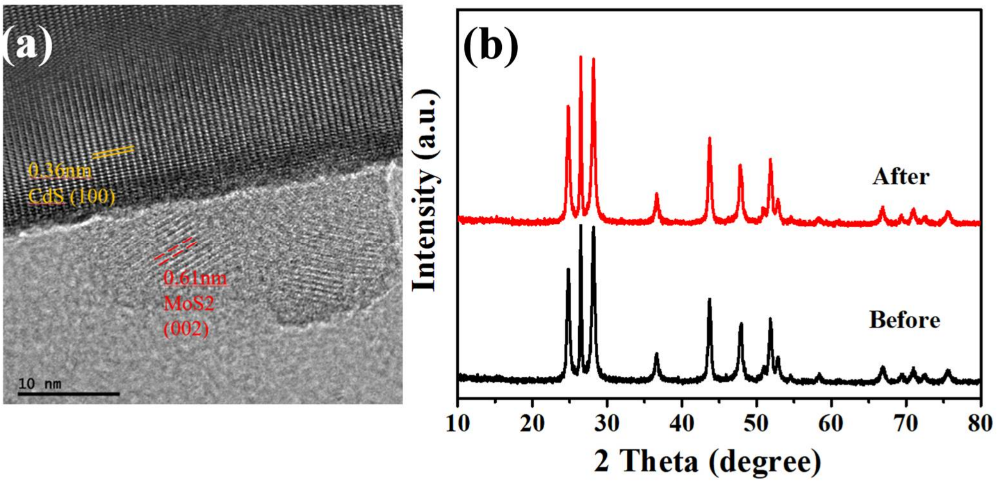

Figure S3. The characterization of CdS/P/MoS_2 after recycling tests: (a) HRTEM; (b) XRD.

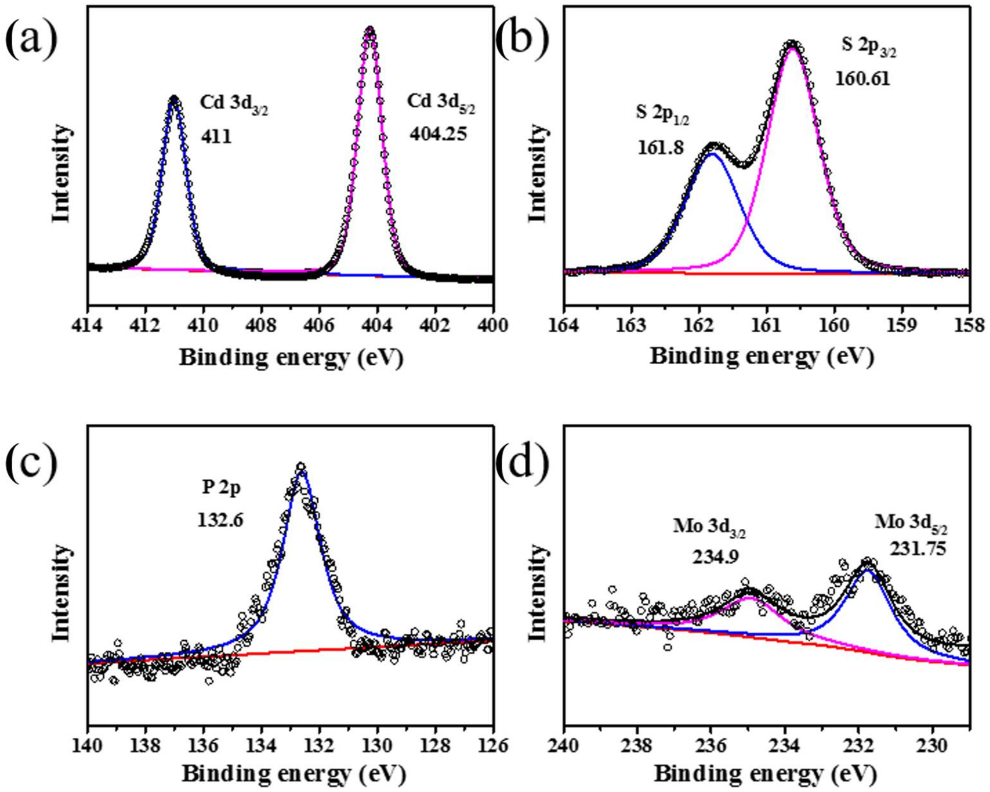

Figure S4. the high resolution XPS spectra of CdS/P/MoS_2 after a photocatalytic run: a) Cd 3d; b) S 2p; c) P 2p and d) Mo 3d.

Figure S3a shows the HRTEM image after photocatalytic test for 12 h, and Figure S3b shows the XRD patterns of CdS/P/MoS_2 before and after photocatalytic test for 12 h. As can be seen from Figure S3, the structure of CdS/P/MoS_2 does not change significantly after the photocatalytic reaction. As the figure S4 shows, there are no changes in the surface species of CdS/P/MoS_2 after a photocatalytic run.This phenomenon confirmed that MoS_2 as a cocatalyst can promote the separation and transfer of photogenerated charge carriers and improve the photocatalytic activity.

Table S1. Comparison of photocatalytic H2 production activities over different photocatalyst.

Photocatalyst	Light (nm)	Activity ($mmol \cdot g^{-1} \cdot h^{-1}$)	Stability (h)	References
Pt/CdS nanorods	>420	4.46	>50	[51]
Pt/CdS/TNT	>420	4.26	>3	[52]
Ru/CdS/ZnS nanoparticles	>420	5.85	>40	[53]
Pd@C_3N_4	>400	53.4	>6	[54]
CdS/FeP	>420	278	>120	[12]
Co/CdS nanorods	>420	14.2	>12	[55]
Ni/CdS nanorods	>420	22.8	>12	[55]
Co-Ni/CdS nanorods	>420	32.6	>18	[55]
CdS/Co-P_3	>420	102.9	>24	[6]
TiO_2/AuPd	solar	17.7	>10	[56]
NiCoP@CdS nanorods	>400	354	>48	[57]
CdS/CoP@RGO	>420	182	>160	[2]
CdS/P/MoS_2	>400	68.89	>12	this work

Table S2. Exponential decay-fitted parameters of fluorescence lifetime for CdS, CdS/P and CdS/P/MoS_2.

Items	CdS	CdS/P	CdS/P/MoS_2
τ_1/ns	0.8341	0.8001	0.8272
A_1	251.081	228.995	154.313
τ_2/ns	9.8339	8.2673	6.6510
A_2	5.403	6.574	0.772
$\tau_{average}$/ns	2.66	2.51	1.05

Through double exponential function fitting and calculation, the average fluorescence life of the sample was calculated, as shown in the table S1. The average life of CdS/P/MoS_2 is 1.05 ns, which is much lower than that of CdS/P (2.51 ns) and CdS/P/MoS_2(2.66ns). This proves that CdS/P/MoS_2 has a higher nonradiative transition rate, which is attributed to the transfer of electrons from CdS/P to MoS_2.
